# Case report: Single infusion of combined anti-CD20 and anti-CD38 monoclonal antibodies in pediatric refractory lupus nephritis

**DOI:** 10.3389/fimmu.2025.1525892

**Published:** 2025-01-28

**Authors:** Decimo Silvio Chiarenza, Raul Mancini, Carolina Bigatti, Gianluca Caridi, Alessandro Consolaro, Valentina Natoli, Gabriele Mortari, Xhuliana Kajana, Francesca Lugani, Marco Gattorno, Gian Marco Ghiggeri, Edoardo La Porta, Gabriele Gaggero, Enrico E. Verrina, Andrea Angeletti

**Affiliations:** ^1^ Nephrology, Dialysis and Transplantation Unit, IRCCS Istituto Giannina Gaslini, Genoa, Italy; ^2^ Translational Transplant Research Center and Department of Medicine, Icahn School of Medicine at Mount Sinai, New York, NY, United States; ^3^ Rheumatology and Autoinflammatory Diseases, IRCCS Istituto Giannina Gaslini, Genova, Italy; ^4^ Dipartimento di Neuroscienze, Riabilitazione, Oftalmologia, Genetica e Scienze Materno-Infantili (DiNOGMI), Università degli Studi di Genova, Genova, Italy; ^5^ Pathology Unit, IRCCS Istituto Giannina Gaslini, Genova, Italy

**Keywords:** systemic lupus erythematous, lupus nephritis, rituximab, daratumumab, monoclonal antibodies, pediatric

## Abstract

Lupus nephritis (LN), present in 30%–50% of systemic lupus erythematosus (SLE) patients, often necessitates standard immunosuppressive therapy (glucocorticoids, MMF, CYC) as suggested by the European League Against Rheumatism/European Renal Association–European Dialysis and Transplant Association (EULAR/ERA-EDTA) and Kidney Disease Improving Global Outcomes (KDIGO) guidelines. However, a subset of subjects remains refractory. Recent findings suggested the efficacy of targeting CD38-long-lived plasma cells in LN and SLE refractory to standard treatment. However, previous experiences were limited to adult patients and described different therapeutical schemes based on daratumumab, with the addition or absence of belimumab. Moreover, the minimal effective dose of daratumumab has yet to be fully defined. In this report, we describe two cases of juvenile-onset refractory LN/SLE successfully managed with a combination of a single infusion of rituximab (targeting CD20 on B cells) and daratumumab (targeting CD38 on long-lived plasma cells), unlike prior regimens requiring prolonged daratumumab infusions. Our approach was safe and effective and may potentially reduce adverse effects and costs, providing a novel therapeutic option for juvenile refractory LN.

## Introduction

Lupus nephritis (LN) is the most common complication in systemic lupus erythematosus (SLE), affecting 30%–50% of patients. Both the European League Against Rheumatism/European Renal Association-European Dialysis and Transplant Association (EULAR/ERA-EDTA) (1) and the Kidney Disease Improving Global Outcomes (KDIGO) guidelines (2) are mostly based on non-specific immunosuppressive agents, including glucocorticoids, mycophenolate mofetil (MMF), or cyclophosphamide (CYC), which represent the standard of care (SOC). However, the SOC may be only partially effective in more complicated cases. Indeed, a consistent minority of patients do not achieve a complete response and are classified as refractory. Refractory SLE/LN is defined by the failure to achieve a complete response within 3-4 months, partial remission within 6-12 months, or complete remission after 48 months of the SOC (3).

SLE is largely considered a typical autoimmune disease, sustained by different circulating autoantibodies targeting several cellular components (4, 5). Therefore, the imbalance of the B cell tolerance may represent a key point in the pathogenesis of SLE (6) and provides a rationale for B cell-targeted therapy, as previously reported. More recently, non-dividing CD38-long-lived plasma cells that, unlike short-lived plasmablasts, reside in dedicated niches in the bone marrow and do not express CD20 on the surface, have been proposed as a relevant target in refractory cases of SLE and LN. In line with this, previous studies on mouse models of SLE reported that antiproliferative immunosuppressive therapy, such as CYC, depleted short-lived plasmablasts, but not long-lived plasma cells, which may continue to produce autoantibodies (7).

Daratumumab is a fully human monoclonal antibody targeting CD38 and represents the cornerstone of treatment in multiple myeloma. In two different case series, Ostendorf et al. (8) and Roccatello et al. (9) have recently described the efficacy of targeting CD38-long-lived plasma cells in LN and SLE refractory to standard treatment. However, previous experience described different therapeutical schemes based on daratumumab, such as the addition or absence of maintaining treatment with belimumab (8). Moreover, the minimal effective dose of daratumumab has yet to be fully defined.

Therefore, herein, we describe two cases of juvenile refractory LN/SLE treated with a combined single infusion of the monoclonal chimeric anti-CD20 antibody rituximab and daratumumab.

## Case presentations

### Patient 1

**Figure 1:**
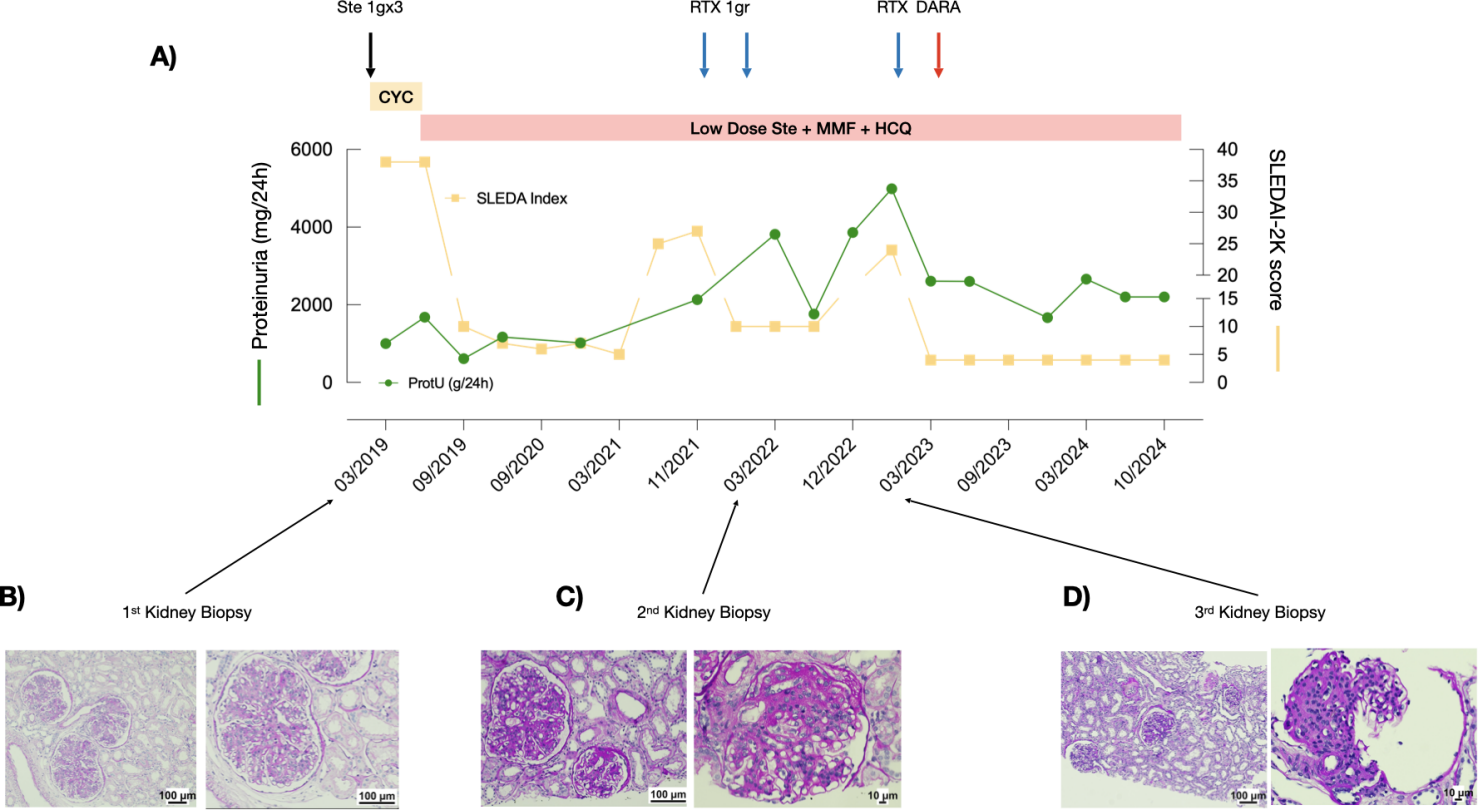
Response to treatment with combined rituximab and daratumumab in Patient 1. **(A)** Proteinuria levels (green line) and SLEDAI scores (yellow line) during the observation period. **(B-D)** PAS staining of the three kidney biopsies performed at recurrence.

Patient 1 is a 17-year-old girl who presented at 14 years of age with low-grade fever, asthenia, malar rash, headache, oral ulcers, arthralgias/arthritis, and pericarditis. Laboratory tests showed leukopenia [3.280/mm^3^, normal values (n.v.) 4,200 - 9,800], hypocomplementemia (C3 89 mg/dL, n.v. 95-180, C4 4 mg/dL, n.v. 15 - 53), high inflammatory markers (VES 68 mm/h, n.v. 0-19), hypergammaglobulinemia, anti-nuclear antibodies (ANAs) (titer 1:320 - positivity >1:40) and anti-dsDNA antibodies (68 IU/mL - positivity>10UI/mL) as well as non-nephrotic proteinuria (1g/day) and microscopic hematuria. Therefore, she was admitted to our Nephrology Unit with a diagnosis of SLE and a Systemic Lupus Erythematosus Disease Activity Index 2000 (SLEDAI-2K) score (10) of 38 (**Figures 1A, B**). The kidney biopsy revealed LN class IV+V, characterized by diffuse mesangial expansion and endocapillary hypercellularity, and early signs of focal and segmental sclerosis (**Figure 1C**). Immunofluorescence (IF) staining was positive for a “full house” pattern (IgA ++, IgG +++, IgM ++, C3 +++, C4 +, C1q ++, glomerular C4d +++). The activity and the chronicity index score were 14 and 2, respectively. Therefore, she was treated with the SOC based on high-dose intravenous (IV) glucocorticoid pulses (methylprednisolone 1g/day for 3 consecutive days) and cyclophosphamide according to the Eurolupus regimen (11), consisting of 500 mg of IV cyclophosphamide every 2 weeks for a total of six administrations. After that, maintenance treatment with MMF, oral glucocorticoids, and hydroxychloroquine was started, leading to complete clinical and serological remission.

After 2 years, she experienced a recurrence of clinical signs, including asthenia, arthritis and headache, accompanied by laboratory findings indicative of LN relapse: lymphopenia (890/mm³), reduced complement component C4 (5 mg/dL), elevated anti-dsDNA antibodies (17 IU/mL), and increased proteinuria (2.5 g/day), with a SLEDAI-2K score of 25. A kidney biopsy largely confirmed the previous histological findings, in accordance with LN class IV+V (**Figure 1B**). To minimize overall exposure to cyclophosphamide, two infusions of rituximab (375 mg/m^2^ 6 weeks apart) were administered, resulting in clinical improvement but with persistence of significant proteinuria (1.7 g/day), low C4 levels (4 mg/dL), and positive anti-dsDNA (15 IU/ml). Fourteen months after rituximab infusions, she presented with a significative worsening of proteinuria (4 g/day) with a SLEDAI-2K score of 18. A kidney biopsy was repeated, demonstrating 3/8 sclerotic glomeruli, while the remaining 5/8 presented with mesangioproliferative and membranoproliferative lesions, with a thickening of the basement membrane, 25%-50% of the glomeruli exhibited fibrosis of Bowman’s capsule, and one glomerulus showed neutrophils within the tuft. Segmental sclerosis characterized 25%-50% of the glomeruli with segmental glomerular microthrombosis. The interstitium and large-size vessels did not present with significant lesions. IF staining showed a partial remission of the “full-house” pattern (IgA ++, IgG +, IgM -, C3 -, C4 -, C1q +), consistent with an activity index score of 5 and chronicity index score of 4 (**Figure 1D**).

In light of the histological findings, despite the overall moderate disease activity, the patient was treated with a combination of a single infusion of rituximab (375 mg/m^2^) and daratumumab (1.2g), with a consequent significant reduction in proteinuria (from 4.9 to 2.6 g/day), improvement of activity disease index, such as complement component C4 (17 mg/dl) and anti-dsDNA antibodies (3.6 UI/ml), and a reduction in SLEDAI-2K score to 4. At 2 years of follow-up, she is receiving standard therapies with low-dose glucocorticoids (prednisone 5 mg/day), MMF, and hydroxychloroquine, and proteinuria is stable at 2.6 g/day, with a SLEDAI-2K score of 4, marking the longest remission period since disease onset (**Figure 1A**). The residual significant proteinuria is consistent with chronic damage (chronicity index score of 4).

All the treatments were well tolerated, with no short- or long-term complications, including hypogammaglobulinemia. Renal function was consistently stable within normal values (0.5 mg/dl, n.v. 0.4-1.2). In **Table 1**, we provide the main circulating lymphocyte subtype analysis before the administration of the combined treatment (t_0_) and at 6 (t_6_) and 12 (t_12_) months of follow-up.

**Table 1:** Main circulating lymphocyte subtypes and immunoglobulins.

	Patient 1			Patient 2		
	t_0_^#^	t_6months_^#^	t_12months_^#^	t_0_^#^	t_6months_^#^	t_12months_^#^
CD19^+^ * (n.v. 6-13)	1.3	0.3	1.1	8.4	2.3	6.2
NK cells * (CD16^+^CD56^+^CD3^+^) [n.v. 3.3-4.6]	0.6	1.2	0.7	1.4	1.2	1.3
CD3^+^CD4^+^ * [n.v. 37-50]	48.7	49.5	49.4	46.3	46.3	48.6
CD3^+^CD8^+^ * [n.v. 20-32]	34.8	31.2	32.7	32.4	32.4	37.3
Plasma Cells * CD38high [n.v. 2-6]	5.2	1.2	4.3	7.8	2.5	5.6
IgG (mg/dl) [n.v. 700-1600]	1105	438	726	1185	513	720
IgA (mg/dl) [n.v. 70-400]	93	107	69	113	94	149
IgM (mg/dl) [n.v. 40-230]	25	12	16	98	32	53

n.v., normal values.

*Expressed as a percentage of total lymphocytes.

#Refers to the combined treatment with rituximab and daratumumab.

### Patient 2

**Figure 2:**
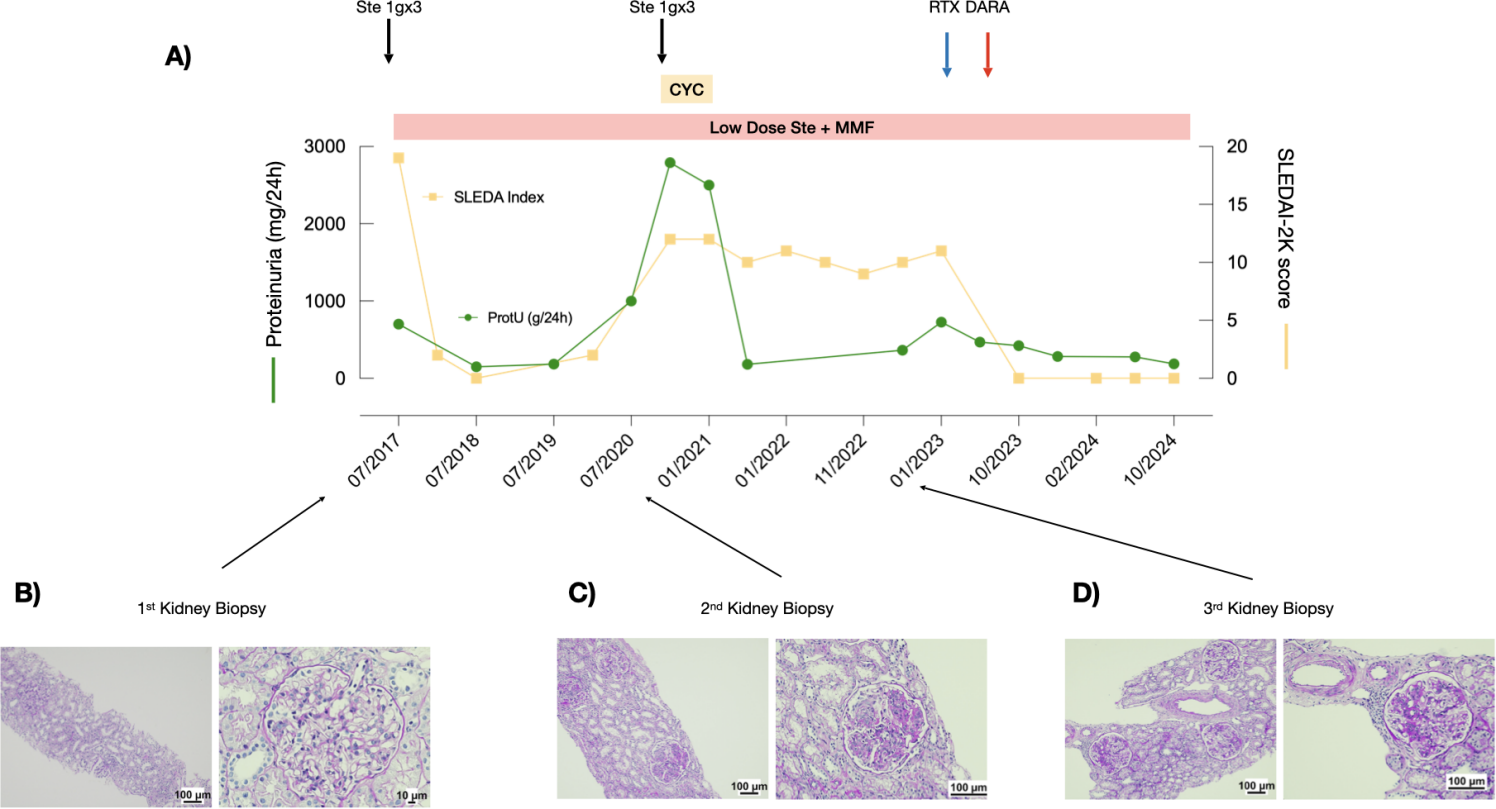
Response to treatment with combined rituximab and daratumumab in Patient 2. **(A)** Proteinuria levels (green line) and SLEDAI scores (yellow line) during the observation period. **(B–D)** PAS staining of the three kidney biopsies performed at recurrence.

Patient 2 is a 22-year-old man who was diagnosed with SLE at 15 years of age after presenting with fever, arthritis, malar rash, chilblains, proteinuria (0.5-0.7 g/day), microscopic hematuria, consumption of serum complement components C3 and C4 (C3 42 mg/dL, C4 6 mg/dL), ANA positivity (1:160), and anti-dsDNA antibodies >240 IU/mL (SLEDAI-2K=19)(**Figure 2A**). A kidney biopsy was performed, revealing mesangial expansion and proliferation (**Figure 2B**). IF staining showed IgA +, IgG +++, IgM -, C3 ++, C4 +, C1q ++, and glomerular C4d ++. The activity and the chronicity index scores were 10 and 2, respectively. He received the SOC including MMF, glucocorticoids, and hydroxychloroquine, with consequent complete clinical and serological remission. After 3 years, he presented with a disease flare, with proteinuria 2.7 g/day and consumption of the complement components (C3 61mg/dL, C4 7 mg/dL) (SLEDAI-2K score was 12). A second kidney biopsy was performed, which showed mesangial and endocapillary hypercellularity in approximately 70%-80% of the glomeruli, with cellular crescents (**Figure 2C**). IF revealed IgA +, IgG +++, IgM +, and C3 +++. The activity and the chronicity index scores were 14 and 2, respectively. Therefore, IV glucocorticoid pulses (1 g/day for 3 consecutive days) and CYC according to the EULAR/ERA recommendations (11) were administered, with remission of the proteinuria. Low-dose glucocorticoids, MMF, and hydroxychloroquine were maintained. However, over the following 2 years, the patient continued to exhibit signs and symptoms consistent with refractory SLE, including headache, photophobia, low complement levels (C3 49-70 mg/dL, C4 6-15 mg/dL), and elevated anti-dsDNA antibodies (169-240 IU/mL) with SLEDAI-2K scores between 10 and 12 (**Figure 2A**), leading to a poor quality of life. Despite this, proteinuria remained negative.

At the 2 year-follow-up after the Eurolupus regimen administration, a third kidney biopsy was performed due to a significant worsening of proteinuria (0.7 g/day): all the glomeruli showed mesangioproliferative and membranoproliferative lesions with a thickening of the basement membrane, which displayed cribriform patterns, double contours, and spikes. Glomerulosclerosis was present in approximately half of the glomeruli (**Figure 2D**). IF revealed deposits of only C3 and C1q (C3 +, C1q ++). His SLEDAI-2K score was 11. The activity and the chronicity index scores were 9 and 2, respectively. Therefore, the patient was treated with a combination of a single infusion of rituximab (375 mg/m^2^) and daratumumab (1g). After the infusions, proteinuria rapidly normalized (0.28 g/day) and laboratory findings improved (C3 112 mg/dl; C4 24 mg/dl). The anti-dsDNA antibody test was negative 1 month after the last infusion.

At the last follow-up, 2 years after daratumumab administration, the patient is receiving standard therapies with low-dose glucocorticoids, hydroxychloroquine, and MMF. Proteinuria remains negative and renal function normal (0.8 mg/dl) and the patient’s SLEDAI-2K score is 0 (**Figure 2A**).

Neither infusion-related reactions, nor short-term adverse events were noted after rituximab and/or daratumumab administration, including hypogammaglobulinemia (**Table 1**).

## Discussion

We recently described the efficacy and the safety of combining treatment with rituximab and daratumumab for complicated forms of podocytopathies both in native kidneys (12) and in post-transplant recurrence in pediatric and young adult subjects (13). Given the favorable safety profile of the therapeutic regimen in pediatric populations, this study reports on the efficacy and safety of combined rituximab and daratumumab treatment in two young adults with refractory class IV and V LN, initially diagnosed during childhood. The administration of daratumumab in SLE/LN was recently reported in two different case series. Ostendorf et al. (8) described two patients affected by life-threatening and refractory SLE with severe multisystemic involvement. The first subject had LN class III-V with nephrotic syndrome, pericarditis, arthritis, and skin rash, despite treatment with MMF, cyclosporine A, and glucocorticoids. The second subject presented with hemolytic anemia, immune thrombocytopenia, cutaneous vasculitis, arthritis, alopecia, and mucosal ulcers, despite previous treatments with cyclophosphamide, MMF, belimumab, rituximab, azathioprine, methotrexate, hydroxychloroquine, plasmapheresis, immunoglobulin, and bortezomib. Both patients were treated with daratumumab (16 mg/kg once a week for 4 weeks), in addition to maintenance treatment consisting of belimumab, low-dose glucocorticoids, and MMF. The four administrations of daratumumab resulted in a significant reduction in anti-dsDNA antibodies and SLEDAI-2K score (8). More recently, Roccatello et al. (9) reported six adult patients with refractory LN treated with daratumumab, according to the multiple myeloma protocol (16 mg/kg once a week for 8 weeks followed by eight biweekly infusions and up to eight monthly infusions). In 5/6 patients, their SLEDAI-2K score significantly decreased, with improvement in kidney function and remission of proteinuria at the 12-month follow-up.

Overall, previous data suggest the potential effectiveness of multiple infusions of daratumumab in refractory LN and SLE (8, 9). We here described the effectiveness of combining a single infusion of monoclonal anti-CD20 with anti-CD38 antibodies in two cases of juvenile SLE, which are usually characterized by higher incidence of renal involvement than patients with adult-onset SLE (14). We did not administer daratumumab alone because rituximab, despite a previous negative clinical trial (15), is already suggested as possible treatment in refractory SLE (16). Therefore, from an ethical standpoint, we decided not to prevent patients from receiving a treatment already recognized as potentially effective.

Therefore, in contrast to previous experiences, we administered only single infusions of rituximab and daratumumab. Indeed, after the dual treatment with rituximab and daratumumab, the remission was maintained with standard therapies consisting with low-dose glucocorticoids, hydroxychloroquine, and MMF in both patients, without the chronic addition of belimumab as reported by Ostendorf et al. (8).

As expected, we report a significant reduction in total CD19+ B cells and circulating CD38+ plasma cells after the combined treatment that fully recovered 12 months after treatment. Different immune phenotyping did not reveal other major changes after treatment (**Table 1**). However, further studies are needed to assess whether daratumumab alone, by depleting CD38+ plasma cells, is sufficient in promoting disease remission.

Overall, this therapeutic approach led to a significant response in both cases with rapid and sustained remission, reducing the need for additional immunosuppressive agents or further infusion of daratumumab, along with their possible side effects and costs. Moreover, both patients confirmed that the stable disease remission led to a significant increase in their quality of life. Generally, the standard treatment for SLE and LN, involving high-dose glucocorticoids and immunosuppressants such as cyclophosphamide, may lead to severe complications such as infections, metabolic issues, and possible organ damage. Achieving sustained remission is crucial to reduce toxic therapies, prevent flares, and slow CKD progression in LN, improving long-term outcomes. Emerging targeted therapies offer the promise of greater efficacy with less toxicity, enhancing patient quality of life and reducing healthcare burdens.

In conclusion, we described the efficacy and the safety of a combined single infusion of rituximab and daratumumab in two young adult subjects with refractory juvenile-onset SLE SLE/LN. Currently, a Phase 2 Open-label trial, testing the efficacy and safety of daratumumab in adult subjects affected by LN, is in the enrolling phase (NCT04868838). However, given the safety profile reported here, and considering that juvenile-onset disease more frequently involves renal manifestations compared to adult-onset (17), future studies should also include pediatric patients with LN and explore whether this approach might also benefit different systemic involvements, such as neuropsychiatric SLE (NPSLE).

## Data Availability

The raw data supporting the conclusions of this article will be made available by the authors, without undue reservation.
